# Nutrigenomic underpinnings of intestinal stem cells in inflammatory bowel disease and colorectal cancer development

**DOI:** 10.3389/fgene.2024.1349717

**Published:** 2024-08-30

**Authors:** Jennifer Ho, Nicholas Puoplo, Namrata Pokharel, Aanya Hirdaramani, Aylin C. Hanyaloglu, Chia-Wei Cheng

**Affiliations:** ^1^ Institute of Human Nutrition, Columbia University Irving Medical Center, New York City, NY, United States; ^2^ Columbia Stem Cell Initiative, Columbia University Irving Medical Center, New York City, NY, United States; ^3^ Division of Neonatology-Perinatology, Department of Pediatrics, Columbia University Irving Medical Center, New York City, NY, United States; ^4^ Department of Metabolism, Digestion and Reproduction, Division of Digestive Diseases, Section of Nutrition, Faculty of Medicine, Imperial College London, London, United Kingdom; ^5^ Department of Metabolism, Digestion and Reproduction, Institute of Reproductive and Developmental Biology, Faculty of Medicine, Imperial College London, London, United Kingdom; ^6^ Department of Genetics and Development, Columbia University Irving Medical Center, New York City, NY, United States

**Keywords:** nutrigenomics, intestinal stem cells, inflammatory bowel disease, colorectal cancer, transcriptional regulation, food-gene interaction

## Abstract

Food-gene interaction has been identified as a leading risk factor for inflammatory bowel disease (IBD) and colorectal cancer (CRC). Accordingly, nutrigenomics emerges as a new approach to identify biomarkers and therapeutic targets for these two strongly associated gastrointestinal diseases. Recent studies in stem cell biology have further shown that diet and nutrition signal to intestinal stem cells (ISC) by altering nutrient-sensing transcriptional activities, thereby influencing barrier integrity and susceptibility to inflammation and tumorigenesis. This review recognizes the dietary factors related to both CRC and IBD and investigates their impact on the overlapping transcription factors governing stem cell activities in homeostasis and post-injury responses. Our objective is to provide a framework to study the food-gene regulatory network of disease-contributing cells and inspire new nutrigenomic approaches for detecting and treating diet-related IBD and CRC.

## 1 Introduction

Connections between diet and human disease processes are well established, yet the precise mechanisms by which nutrition affects human health remain largely speculative. Nutrigenomics and nutrigenetics are emerging fields that study the relationship between nutrient intake and genetics. While nutrigenetics explores individual genetic variations, nutrigenomics examines how nutrient intake can regulate gene expression (Mead, 2007; Kiani et al., 2022). This review focuses on the nutrigenomics of intestinal stem cells (ISCs), examining how specific nutrients alter gene expression to modulate ISC function and activity. Specifically, we aim to provide a comprehensive analysis of the nutrigenomic effects on ISCs and their influence on the development of inflammatory bowel disease (IBD) and colorectal cancer (CRC). Our objectives are to identify dietary factors linking the pathogenesis of IBD and CRC, with a particular focus on their effects on the transcriptional programs that regulate ISC activity through gene expression (i.e., stem cell nutrigenomics). We will review human studies to narrow down dietary factors with strong disease associations and examine the mechanistic connections between the identified dietary factors, transcriptional programs, stem cell responses, and disease-related phenotypes in pre-clinical models. This review will synthesize current evidence on dietary regulation of stem cell function and provide a comprehensive understanding of how key food-gene interactions alter ISC behaviors and contribute to IBD and CRC.

## 2 Human epidemiologic evidence for dietary association to both CRC and IBD

Human epidemiologic data indicate a strong association between gastrointestinal diseases and dietary patterns when considering incidence, progression, as well as prevention and alleviation. CRC and IBD exemplify this relationship, though data directly linking diet to IBD is limited and is an active field of investigation.

CRC is the third most commonly diagnosed cancer worldwide and the second most common cause of cancer-related deaths (Duan et al., 2022; Siegel et al., 2023). Most CRC cases begin with the development of abnormal crypts, leading to the formation of a neoplastic precursor polyp that eventually evolves into cancer over 10–15 years (Dekker et al., 2019). Although genetic factors play a role in CRC epidemiology, 60%–65% of cases arise sporadically due to lifestyle and nutritional patterns (Keum and Giovannucci, 2019). Specifically, one systematic review found that “Western” dietary patterns comprised of red and processed meat, refined grains, and high-fat dairy were significantly associated with an increased risk of CRC when compared to diets composed of fruits, vegetables, low-fat dairy, and fish (Garcia-Larsen et al., 2019). A prospective study published in 2020 using the UK biobank revealed a 20% higher risk of CRC development in individuals who ingested 76 g of red or processed meat per day compared to 21 g (UK government recommendation is < 90 g/day) (Bradbury et al., 2020). Alcohol intake was associated with an 8% increased risk of CRC for every 10 g ingested daily (about ½ of a pint of 4.5% beer). Those in the highest fifth of fiber intake from breads and cereals had a 14% decrease in CRC risk. Though human epidemiologic studies naturally have limitations, this study was large and adjusted for confounding factors. As of 2018, there were 1.8 million new CRC cases diagnosed annually, with escalating rates in less affluent countries (Keum and Giovannucci, 2019). The expansion of the Western-style diet may be a major cause of increasing global cases of CRC as the Western lifestyle becomes more widely adopted. It is important to note that the Western-style diet encompasses many nutritional factors that individually have an influence on disease, which we will explore further below.

IBD is a chronic, relapsing inflammatory disorder of the intestine, primarily manifesting as ulcerative colitis (UC) and Crohn’s disease (CD). These diseases differ in various ways, including inflammatory depth and location; CD causes transmural inflammation at any portion along the GI tract, whereas UC is limited to mucosal inflammation in the colon. The etiology of IBD remains unknown, but it likely involves a combination of genetic predisposition and environmental factors, including diet, though the association is not as clear as it is with CRC. Approximately 8%–12% of patients with IBD have a positive family history (Jarmakiewicz-Czaja et al., 2022), though this factor alone does not account for most cases (El Hadad et al., 2024).

The connection between IBD and CRC is well documented, with IBD patients facing double the risk of developing CRC compared to the general population (Nardone et al., 2023). This unique disease entity is termed inflammatory bowel disease-related colorectal cancer (IBD-CRC). In population-based cohorts, a younger age at the onset of IBD is associated with an increased risk of CRC development (Jess et al., 2012; Gausman et al., 2020). For patients with UC, CRC risk increases with illness duration, extent of colitis, and association with other inflammatory disorders, such as primary sclerosing cholangitis. Conversely, anti-inflammatory medications, such as steroids or mesalamine, reduce CRC incidence (Ullman and Itzkowitz, 2011; Sloka et al., 2023). The prevailing understanding is that chronic inflammation predisposes tissue to tumor formation and provides the microenvironment conducive to tumorigenesis. Importantly, a recent systematic review examining the role of nutrition in IBD-CRC pathogenesis and prevention found that diets rich in nutrient density through fruits, vegetables, healthy fats (n-3 polyunsaturated fatty acids (PUFAs) and olive oil), and probiotics, while low in animal protein and processed foods, may be beneficial in preventing inflammation and IBD-CRC (Cassotta et al., 2023). The impact of nutrigenomics on IBD-CRC is an avenue worth exploring. Table 1 provides an overview of human epidemiologic studies that offer evidence for dietary influence on disease predisposition.

**TABLE 1:** Details of noted human epidemiologic evidence for dietary association to both CRC and IBD.

Dietary components	Human epidemiologic evidence CRC	Human epidemiologic evidence IBD
Dietary Fat	Seyyedsalehi et al. (2022) – Multicenter case-control study consisting of 4149 participants from the IROPICAN study in Iran. Food frequency questionnaires (FFQ) were completed from 865 cases (patients with CRC) and 3206 controls. A positive association was found between CRC and high intake of dietary total fat (odds ratio (OR) highest quartile _Q4_ = 1.77, 95% confidence interval (CI) = 1.32–2.38), cholesterol (OR_Q4_ = 1.58, 95% CI = 1.22–2.05), and palmitoleic acid (OR_Q4_ = 2.16, 95% CI = 1.19, 3.91), and an inverse association with high intake of dietary heptanoic acid (OR_Q4_ = 0.33, 95% CI = 0.14, 0.79) and low intake of palmitic acid (OR lowest quartile _Q2_ = 0.53, 95% CI = 0.31–0.88). Wan et al. (202) – Prospective cohort study consisting of 65,550 women from the Nurses’ Health Study (1986-2014) and 45,684 men from the Health Professionals Follow-up Study (1986-2014). FFQs were performed every 4 years. During 2,705,560 years of human year follow-up, 2276 cases of CRC were confirmed. Positive association between CRC risk and intake of monounsaturated fat from animals (hazard ratio (HR) comparing extreme quintiles 1.23; 95% CI 1.02, 1.49; *p* = 0.02 for trend). This association was attenuated when adjusting for red and processed meat consumption. No associations were found for other types of dietary fat or individual fatty acid intake and CRC. Kim and Park (2018) – Systematic review and meta-analysis assessing studies associating dietary fat and CRC. 18 studies were identified. The pooled relative risk with 95% CI for the risk of CRC were 1.00 (CI: 0.90–1.12), 0.97 (CI: 0.86–1.10), 1.08 (CI: 0.92–1.26), and 0.99 (CI: 0.93–1.04) for total fat, saturated fatty acid, monounsaturated fatty acid, and polyunsaturated fatty acid, respectively.	Ananthakrishnan et al. (2014) – Prospective cohort study consisting of 170, 805 women from the Nurses’ Health Study cohort. Diet was assessed every 4 years using FFQs. There were 269 incident cases of CD and 338 incident cases of UC over 26 years. Cumulative energy-adjusted intake from dietary fat, saturated fats, unsaturated fats, n-3 and n-6 polyunsaturated fatty acids (PUFAs) were not associated with risk of UC or CD. However, increased intake of long chain n-3 PUFAs trended towards lowering risk for UC and increased intake of trans-unsaturated fatty acids trended towards increasing UC risk. Hou et al. (2011) – Systematic review of 19 studies. Three studies examined total dietary fat intake and CD risk with two showing positive associations and one reaching statistical significance. Two studies noted high monounsaturated fatty acid (MUFA) intake associated with CD risk with one reaching significance. Two studies noted high PUFA and omega-3 fatty acid intake with CD risk with one reaching significance. Two studies noted high omega-6 fatty acid intake with CD risk with one reaching significance. Notably, three studies examined pre-illness intake of total dietary fat, saturated fat, MUFAs and PUFAs and no differences were found. Five studies noted a positive association between high total dietary fat and UC risk with one reaching significance. Four studies associated MUFAs with UC risk with three reaching significance. Four studies associated high PUFA intake with UC risk with two reaching significance. Notably, two studies found no significant difference in pre-illness intake of total fat, saturated fat, MUFAs or PUFAs in UC patients vs. controls.
Dietary Sugar	Joh et al. (2021) – Prospective cohort study of adolescent dietary information from Nurses’ Health Study II including 33,106 participants using FFQs. During follow-up, 2909 conventional adenomas (758 high risk) and 2355 serrated lesions were identified. High sugar and sugar sweetened beverage intake was associated with adenoma risk but not associated with risk of serrated lesions. For each 5% increment of calories from fructose intake, multivariate ORs were 1.17 (95% CI 1.05–1.31) for total adenomas and 1.30 (95% CI 1.06–1.60) for high-risk adenomas. This risk was only noted for intake during adolescence, not adulthood. Hur et al. (2021) – Prospective cohort study utilizing the Nurses’ Health Study II including 95,464 women with FFQs completed every 4 years. 109 incident early-onset CRC cases were noted and women who had ≥2 servings/day had a more than doubled risk of early-onset CRC (relative risk (RR) 2.18; 95% CI 1.10–4.35), with a 16% higher risk (RR 1.16; 95% CI 1.00–1.36) per serving/day increase compared to women with <1 serving/week. Adolescents had increased risk of early-onset CRC with each serving/day increment of increased sugar sweetened beverage intake (RR 1.32; 95% CI 1.00-1.75). Pacheco et al. (2019) – Prospective cohort study utilizing the California Teacher’s Study, including 99,798 women who completed a FFQ at baseline and were followed. At 20 years of follow-up, 1318 incident cases of CRC were identified. Among women consuming ≥1 sugar sweetened beverage per day, there was no significant increased risk of CRC found compared to non-consumers (HR 1.14 (CI 95% 0.86, 1.53)).	Fu et al. (2022) – Prospective cohort study including 121,490 participants in the UK Biobank with repeated 24-h diet recalls over a 3-year period. During a mean follow-up of 10.2 years, 143 incident UC cases and 367 incident CD cases were documented. An association was made for those who drank >1 sugar sweetened beverage per day compared to non-consumers (HR 1.51, 95% CI 1.11–2.05). Hou et al. (2011) – Systematic review of 19 studies. Five studies reported on total carbohydrate intake and CD risk and no studies found associations. When examining monosaccharides and disaccharides, two studies found associations of increased intake with CD risk and one reached significance. One study found an association between polysaccharide intake and CD risk (OR 5.5, 95% CI 2.1-14.2). Three studies reaching significance noted increased mono- and disaccharide intake in pre-illness CD patients compared to controls. Six studies examined total carbohydrate intake and UC risk but no associations were found. Three of four studies noted associations between monosaccharide and disaccharide intake and UC risk with two reaching significance. No associations were found for polysaccharide intake and UC risk.
Fiber	Aune et al. (2011) – Systematic review and meta-analysis of 25 prospective observational studies. It was found that a high intake of dietary fiber, particularly cereal fiber (8 studies, CI 0.83-0.97) and whole grains (6 studies, CI 0.78-0.89), was associated with a reduced risk of CRC. Bradbury et al. (2020) – Prospective cohort study utilizing the UK biobank consisting of men and women ages 40-69, of which 175, 402 filled out multiple dietary assessments after an initial FFQ. Subjects were followed for an average of 5.7 years in which 2609 CRC cases occurred. Participants in the highest fifth of fiber intake from bread and breakfast cereals had a 14% lower risk of CRC.	Fritsch et al. (2021) – Parallel, cross-over study of 17 patients with UC in remission or mild-disease, randomized to either a low-fat and high-fiber diet or an improved standard American diet. Patients in both groups benefitted from a healthier diet compared to baseline; however, the low-fat and high-fiber group had decreased markers of inflammation (CRP, amyloid A) and decreased dysbiosis (decreased Actinobacteria, increased *Bacteroides*). Hou et al. (2011) – Systematic review of 19 studies. Five studies examined the association between fruit, vegetables and fiber intake with disease risk. All five studies showed associations with two studies reaching significance for decreased CD risk with increased fruit intake. Three studies examined the association between fiber directly and CD risk with one study reaching significance in which those who consumed 22.1 g/d of fiber were at decreased risk compared to those who consumed 12.8 g/d (OR 0.12, CI 0.04-0.37). Notably, for UC, of 8 studies which examined the relationship between fruit, vegetables and fiber, none of the associations reached statistical significance.
Vitamin D	Li et al. (2023) – Prospective cohort study utilizing the UK biobank study consisting of 403,170 participants examining the association between serum 25(OH)D and CRC risk. During 4,957,677 person-years of follow-up, 5053 incident cases were reported. It was found that those with higher serum 25(OH)D levels were at significantly decreased risk of CRC incidence in a dose-dependent manner with a HR of 0.94 (95% CI 0.91-0.97) per 1 standard-deviation increase in serum 25(OH)D level.	Vernia et al. (2022) – Systematic review stating that mean vitamin D levels are lower in IBD patients than the general population. Independent risk factors for vitamin D deficiency include high BMI (>30 kg/m^2^) with CD or UC and IBD-related surgery in CD. In terms of disease activity, those with low levels of serum 25(OH)D (<25 ng/mL) had increased identification of endoscopic and histologic activity. Those with levels >30 mg/mL had a reduced postoperative rate of endoscopic recurrence after CD surgery (OR 0.22, 95% CI 0.07-0.66).

## 3 Nutritional control of ISC trajectories in IBD and CRC development

**FIGURE 1:**
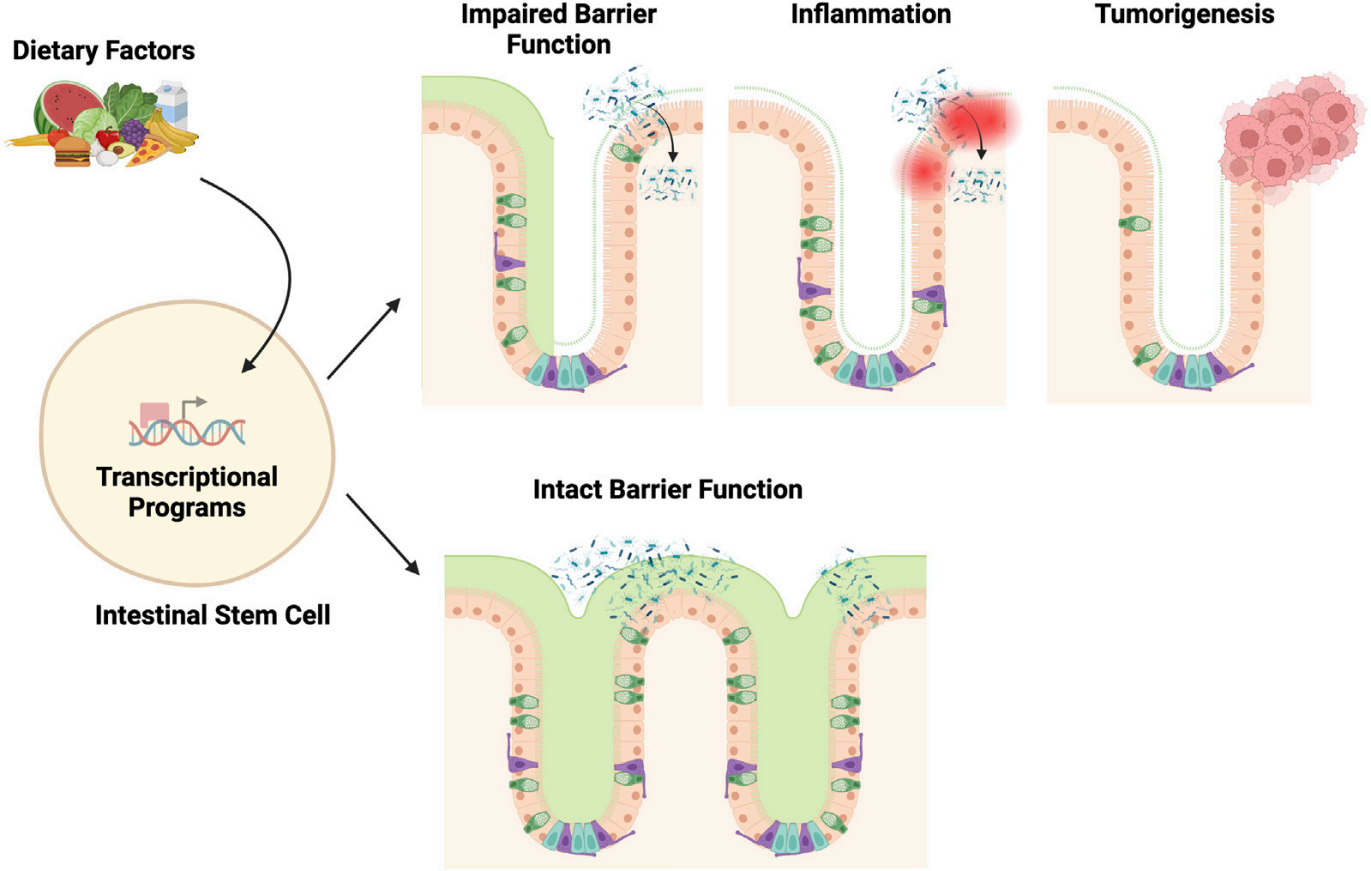
Stem cell nutrigenomics. This broad overview of nutrigenomics describes how dietary choices alter stem cell gene transcription through cell signaling and transcription factors. Certain nutrients (vitamin D, fiber) will lead to healthy stem cell functioning and the maintenance of an intact gut barrier. Other nutrients (dietary fat, sugar) may lead to altered barrier function, inflammation, and possible tumorigenesis.

Although it remains challenging to experimentally depict dietary responses in disease-contributing cells at the transcriptional level in human studies, mechanistic research using genetic mouse models has provided highly translatable insights into how diet and nutrition may influence ISC trajectories. These studies suggest that dietary factors can alter stem cell signaling and its downstream transcriptional activities, potentially contributing to IBD and CRC development (Figure 1). In the following, we will summarize the dietary factors common to both IBD and CRC and explore the corresponding stem cell signaling pathways involved in tissue maintenance, regeneration, and disease development. By integrating these findings, we aim to provide a comprehensive view of the nutrigenomic underpinnings of ISCs in the development of IBD and CRC, which will help inspire new diagnostic and therapeutic approaches.

**FIGURE 2:**
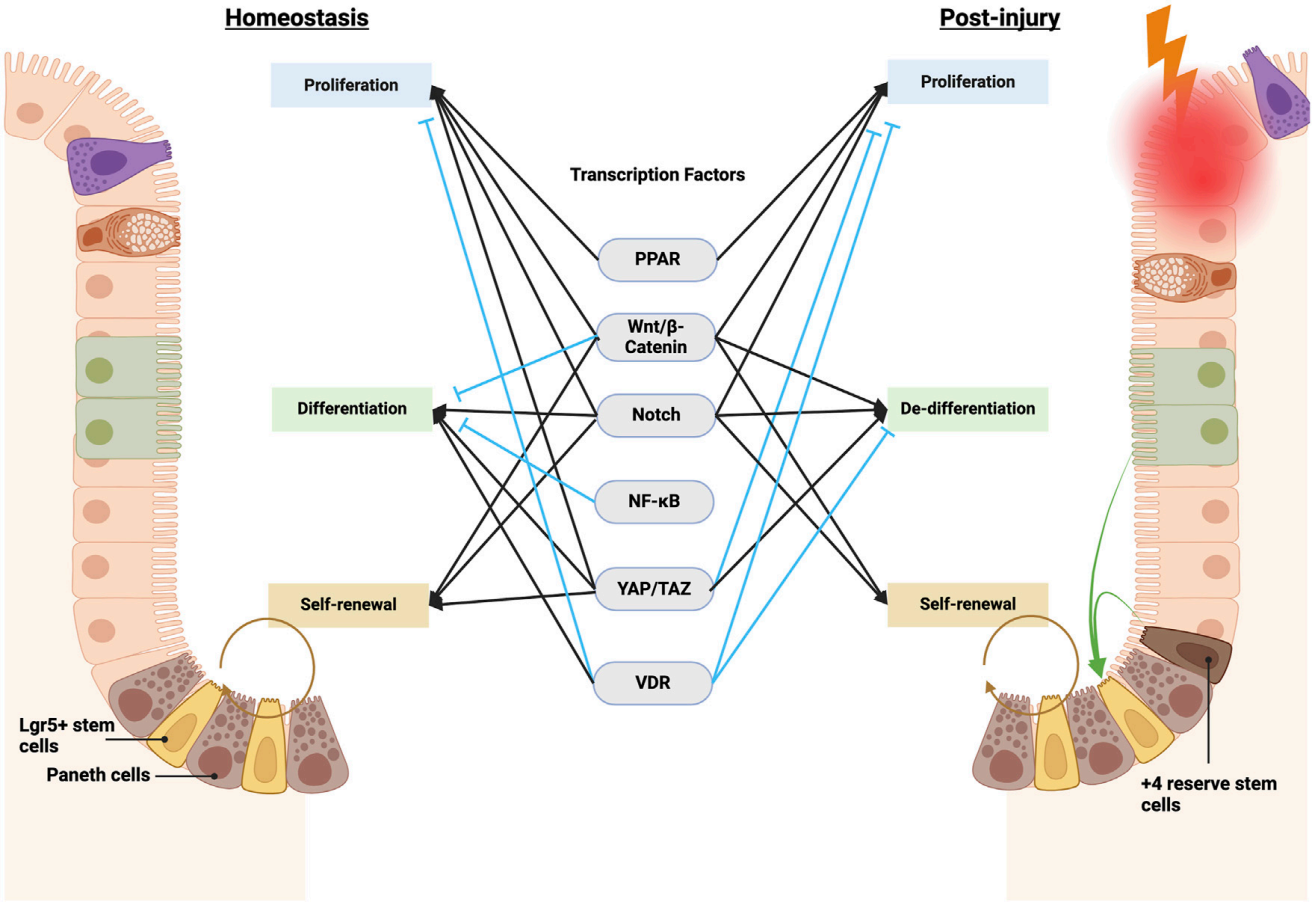
Transcription factors governing ISC function in homeostasis and post-injury regeneration. Key transcription factors, including Wnt, Notch, and Hippo, govern ISC equilibrium in homeostasis by calibrating self-renewal, differentiation, and proliferation. Other transcription factors, such as NF-κB, integrate environmental cues. Injury shifts control–Wnt and Notch promote dedifferentiation and suppress maturation, while altered Hippo activity also restrains aberrant Wnt-mediated hyperproliferation, together enabling regeneration. NF-κB further assists proper ISC differentiation by intersecting with Wnt and Notch. Overall, coordinated signaling integration preserves intestinal homeostasis yet adapts to maximize injury-induced reparative capacity. Elucidating specific contextual pathway utilization remains vital to inform therapeutic modulation. Notably, many homeostatic and post-injury pathways directly alter ISC transcription through transcription factors and activators, including PPAR, Wnt/β-catenin, Notch, NF-κB, YAP/TAZ, and VDR (vitamin D receptor). See details in Supplementary Table 1.

**FIGURE 3:**
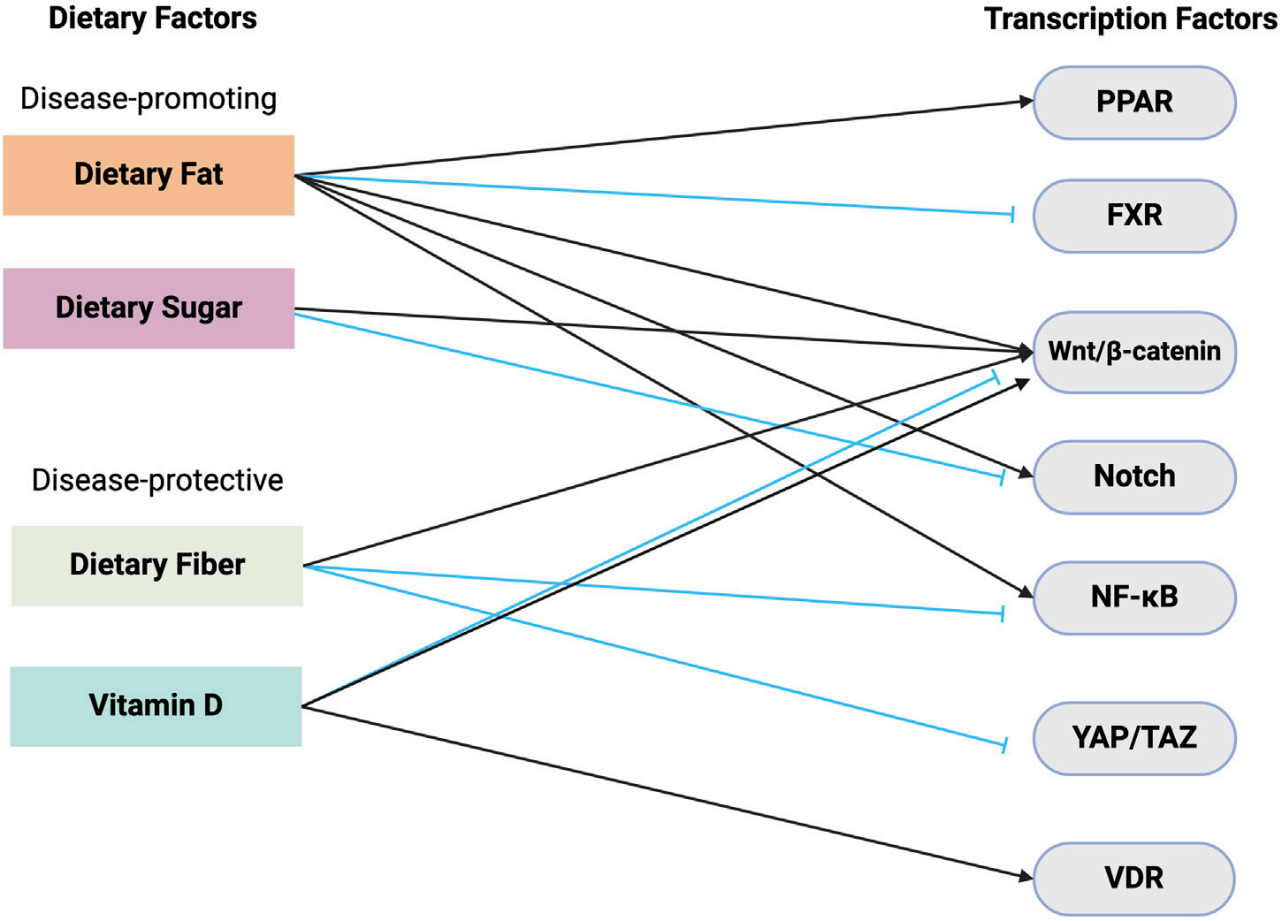
Dietary relation to stem cell transcription. This schematic illustrates the complex interplay between dietary components and its effects on key transcription factors responsible for ISC activity. Dietary fat and sugar are shown as disease-promoting factors. Dietary fat leads to the activation of transcription factors such as PPAR, β-catenin, and Notch, and the inhibition of FXR. Dietary sugar also increases β-catenin signaling but inhibits Notch. In contrast, dietary fiber and vitamin D are depicted as disease-protective components. Dietary fiber reduces the activity of NF-κB and YAP/TAZ transcription factors. Vitamin D exerts its protective effects through its interaction with VDR and disease-specific activation/inhibition of β-catenin signaling. Black arrows = activation, blue arrows = inhibition. Abbreviations: VDR, Vitamin D receptor; NF-κB, Nuclear factor kappa B; PPAR, Peroxisome proliferator-activated receptor; FXR, Farnesoid X receptor; YAP/TAZ, Yes-associated protein/transcriptional co-activator with PDZ-binding motif.

Numerous pre-clinical studies have demonstrated that ISC function, including barrier function and regenerative function, is altered through signaling pathways triggered by specific nutrients and metabolites. Often, these signaling pathways directly alter gene expression through transcription factor regulation (Supplementary Table 1, Figure 2). The precise regulation of ISC behavior is necessary for maintaining the integrity and function of the epithelium barrier. Diet-induced transcriptional alterations that perturb ISC-mediated intestinal homeostasis and post-injury regeneration may predispose the intestinal epithelium to gastrointestinal diseases such as IBD and CRC (Figure 3). Investigations into the intricate interplay between ISCs and the development of CRC and IBD have unveiled a profound connection between ISC behavior and the pathogenesis of these gastrointestinal disorders. Particularly, recent studies have elucidated a nutrigenomic regulatory network, revealing that specific diets and dietary patterns modulate key signaling pathways and subsequent transcriptional activity that determine the cell fate of ISCs in homeostasis, post-injury regeneration, and CRC development (Huels and Sansom, 2015; Testa et al., 2018; Barker et al., 2009; Calibasi-Kocal et al., 2021; Shi and Wang, 2024; Li et al., 2019). Interestingly, many of these dietary factors and signaling regulations also have a strong influence on the trajectories of ISC in chronic inflammation and IBD development.

### 3.1 Dietary factors promoting CRC and IBD

#### 3.1.1 Dietary fat

Diets high in fat (HFD) are commonly associated with an increased risk of both CRC and IBD, though large epidemiologic studies have shown mixed results. The biological plausibility for a HFD leading to CRC is vast but includes obesity-related chronic inflammation, insulin resistance, hormone imbalances, bile acid deposition in the colon, and an altered gut microbiome (Karunanithi and Levi, 2018). A prospective study utilizing the Nurses’ Health Study cohort and the Health Professionals Follow-up Study cohort was recently published, examining associations between fat intake and CRC development. A positive association was found between monounsaturated fatty acid (MUFA) intake from animals and CRC development. It was noted that this association was attenuated when accounting for red and processed meat (Wan et al., 2022). Other large studies, including a case-control study in Iran, found associations between certain fat-related dietary components (total dietary fat, cholesterol, palmitoleic acid, and inverse associations for high heptanoic acid intake and low palmitic acid intake) and CRC when followed over time, though these results stem from food frequency questionnaires (FFQs) and confounding variables are inevitable (Seyyedsalehi et al., 2022). This study found a ∼60% increase in CRC risk with high dietary fat intake. On the contrary, a large meta-analysis found no associations between intake of total fat, saturated fat, MUFAs, or PUFAs and CRC risk (Kim and Park, 2018). Similarly, when considering IBD, a prospective cohort study examining cumulative energy-adjusted intake of total fats, saturated fats, unsaturated fats, and n-3 and n-6 PUFAs found no association with an increased risk of UC or CD (Ananthakrishnan et al., 2014). A systematic review assessing dietary habits and IBD risk found an association with increased fat intake and both UC and CD risk (Hou et al., 2011).

Fatty acid components and/or their derivatives have been shown to influence ISC function in homeostasis, post-injury regeneration, inflammation, and tumorigenesis. HFD-fed mice exhibits β-catenin activation, increases ISC number, and proliferation of progenitor cells (Mao et al., 2013). These changes are reversible through the use of the β-catenin inhibitor JW55 in HFD-fed mice, demonstrating the pivotal role of the β-catenin signaling pathway in diet-induced changes in the intestinal epithelium (Mao et al., 2013). HFD also enhances crypt regeneration after irradiation, with increased surviving and proliferating Ki67+ crypt cells compared to control (Mana et al., 2021). Regarding inflammation, HFD compromises colonic barrier function, as evidenced by decreased expression of goblet and Paneth cell numbers, reduced tight junction proteins, and increased inflammatory factor expression (Xie et al., 2020; Lee et al., 2017). Notably, the reduced goblet cell expression correlates with Notch activation. Furthermore, Paneth cell antimicrobial activity is also diminished, increasing the vulnerability of the intestinal barrier (Lee et al., 2017). Pro-inflammatory signaling induced by HFDs, particularly through the stimulation of cytokines such as TNFα and subsequent activation of NF-κB, can impair tight junction expression and structure (Rohr et al., 2020). In terms of mucosal integrity, HFDs appear to affect mucus layer integrity by reducing goblet cell abundance and altering mucin production (Gulhane et al., 2016). The mucus layer provides a key immunological barrier, and its disruption enables inflammation. HFD further elevates CRC-associated inflammatory cytokine production alongside heightened β-catenin and transcription of the downstream Wnt pathway proto-oncogene, *c-myc*. Indirectly, dietary fat may exert its effects on ISCs through bile acids, which suppress the transcription factor farnesoid X receptor in *Lgr5+* ISCs and induce DNA damage associated with CRC (Fu et al., 2019). Directly, the signaling actions induced by dietary fat are best characterized through the activation of peroxisome proliferator-activated receptor delta (PPAR-δ) (Mana et al., 2021; Beyaz et al., 2016). The heightened PPAR-δ activation evokes expression of a subset of Wnt/β-catenin target genes, such as *Bmp4* and Notch ligands JAG1 and JAG2, in ISCs and progenitors, and promotes both the self-renewal and tumor-initiating abilities of ISCs. Importantly, it also induces the organoid-forming and tumor-initiating ability of the progenitor cells, which otherwise is an exclusive property of ISCs (Xie et al., 2020; Beyaz et al., 2016). This suggests that under HFD conditions, ISCs and progenitors serve as an alternate source of Notch ligands JAG1 and JAG2, thereby acquiring a level of niche independence (Cangelosi and Yilmaz, 2016). In addition to tumor development, the PPAR signaling pathway has also been implicated in the epithelial-mesenchymal transition (EMT) process in cancer stem cells (Zhang et al., 2016; Silva et al., 2021). Elevated Notch signaling has also been observed in both nonmalignant overweight individuals and CRC patients. This trend is replicated in mouse models of HFD, with subsequent administration of the Notch inhibitor DBZ or DAPT showing resistance to HFD-induced obesity, suggesting a potential connection between obesity-induced Notch signaling and CRC development. These findings indicate excessive dietary fat may induce DNA damage, promote ISC self-renewal and progenitor dedifferentiation, and, together, increase susceptibility to tumorigenesis through the activation of Wnt/β-catenin (Beyaz et al., 2016; van der Flier and Clevers, 2009). Notably, n-3 fatty acids are activators of GPR120/FFAR4, which regulates progenitor cell proliferation through Wnt/β-catenin, among other functions. Downregulation of GPR120/FFAR4 has been linked to increased tumorigenesis (Rubbino et al., 2022). It is important to note that PUFAs are not typically thought to be the offending fats in a HFD.

#### 3.1.2 Dietary sugar

An increased incidence of both CRC and IBD has been independently found to be associated with sugar-sweetened beverage intake. In respect to CRC, a prospective cohort study examined the relationship between sugar intake during adolescence and later CRC precursor development (Joh et al., 2021). It was found that in the patients who developed adenomas, high sugar intake and high sugar-sweetened beverage intake during adolescence were associated with adenoma development. Interestingly, this association was not found in adult populations when high sugar intake and sugar-sweetened beverage intake were increased, indicating that high sugar intake during the adolescent years may uniquely increase the risk of future adenoma development. Another prospective cohort study further examined the association between sugar-sweetened beverages and early-onset CRC (Hur et al., 2021). Compared to those who drank ≤1 sugar-sweetened beverage per day, those who drank ≥2 sugar-sweetened beverages per day were at a 2.2-fold higher risk of early-onset CRC. Conversely, replacing sugar-sweetened beverages with artificially sweetened beverages, coffee, or milk reduced the risk of developing early-onset CRC. Notably, not all studies have shown an association between sugar-sweetened beverages and CRC, and thus larger, more diverse, and controlled studies are needed before strong recommendations are made (Pacheco et al., 2019). IBD incidence is also associated with sugar-sweetened beverage intake. A large prospective study found that, compared to non-consumers, those who consumed >1 sugar-sweetened beverage per day had an increased risk for IBD, particularly CD (Fu et al., 2022). Those who consumed natural juices or artificially sweetened beverages did not follow that same association. The authors concluded that sugar-sweetened beverages were associated with IBD development. In the systematic review mentioned earlier examining dietary habits and IBD risk, mixed results were found when examining carbohydrate intake and IBD risk, with some studies showing significant associations (Hou et al., 2011). Limitations of these results include the inherent biases in recall studies.

The effects of dietary sugar on ISC and progenitor activities have been well characterized in mice. In homeostasis, glucose supplementation compromises murine ISC self-renewal and favors lineage-biased differentiation toward secretory fate via inhibition of ketone body-mediated Notch signaling (Cheng et al., 2019). In the setting of the DSS-induced regeneration model, dietary sugar impairs the regenerative functions of crypt ISCs and transit-amplifying cells by inducing ISC stemness and reducing the proliferative potential of *Lgr5+* ISCs (Burr et al., 2023). The sugar-induced impairment in epithelial regeneration is also associated with Notch inhibition (Burr et al., 2023). Regarding inflammation, a high-sugar diet compared to a chow diet exhibits increased vulnerability to experimental colitis and pro-inflammatory cytokines, a compromised gut barrier, decreased microbial diversity, and reduced levels of short-chain fatty acids (SCFAs) (Laffin et al., 2019). Microbial-generated SCFAs rely on the gut microbiota, which can regulate immune and barrier function through suppression of NF-κB (Parada Venegas et al., 2019). Notably, the sugar-induced Notch inhibition in ISCs has yet to be investigated in the context of CRC. Instead, hyperglycemia has been shown to enhance CRC-associated Wnt signaling by targeting the nuclear accumulation of β-catenin (Chocarro-Calvo et al., 2013).

### 3.2 Dietary factors preventing CRC and IBD

#### 3.2.1 Dietary fiber

Dietary fiber, a fundamental component for maintaining gut health, is a nutritional component that has independently been found in various studies to prevent or alleviate both IBD and CRC. A systematic review and meta-analysis showed a decreased risk of CRC development with the ingestion of 10 g of total dietary fiber daily (Aune et al., 2011). The benefit was strongest for fiber from cereal and whole grains and less for fiber from fruits and vegetables. This finding agrees with the aforementioned study from Bradbury et al., in which intake of fiber from bread and breakfast cereals was associated with a reduced risk of CRC (Bradbury et al., 2020). Human studies have also shown that patients with IBD benefit from a high-fiber diet. In a crossover study of patients with UC, biomarkers of inflammation and intestinal dysbiosis were much lower in the fecal samples of patients who ate a low-fat and high-fiber diet when compared to patients who ate an improved standard American diet (Fritsch et al., 2021). Though limited by a small sample size, this study exemplifies further that dietary changes can create measurable improvements in inflammatory states. In an earlier mentioned systematic review, it was found that high fiber and fruit intake are associated with a decreased risk of CD, and high vegetable intake was associated with a decreased risk of UC (Hou et al., 2011). It should be noted that not all studies have demonstrated the benefits of dietary fiber for IBD and CRC, and this association is actively being investigated.

Dietary fiber has been investigated for its function in maintaining proper ISC function. Soluble fiber has been shown to substantially impact the proliferative capacity of *Lgr5+* ISCs in homeostasis (Correa et al., 2023). Soluble fibers are fermented by the gut microbiome into SCFAs such as butyrate, acetate, and propionate, which enhance ISC proliferation and crypt cell turnover *in vivo* (Sakata and von Engelhardt, 1983; Lupton and Kurtz, 1993; Zhang and Lupton, 1994; Sakata, 1987; Edwards et al., 1992; Mathers et al., 1993; Ichikawa et al., 2002; Xie et al., 2022; Salvi and Cowles, 2021; Xie et al., 2024). In addition to activating the GPCRs GPR43/FFAR2 and GPR41/FFAR3, butyrate and propionate can exert potent effects on gene expression via histone deacetylase inhibition. In particular, butyrate downregulates the expression of genes involved in the NF-κB and Hippo signaling pathways in *Lgr5+* intestinal epithelial cells, which might also underlie its anti-proliferative effects *in vitro* (Grouls et al., 2022). In addition, butyrate inhibits epithelial proliferation in a Wnt-5a-dependent manner in mice (Uchiyama et al., 2016). In addition to the effects on ISC proliferation, SCFAs also upregulate the expression of markers indicative of differentiation of *Lgr5+* mouse and human intestinal epithelial cells into secretory cell fates *in vitro*, although exact mechanisms have not been elucidated (Grouls et al., 2022; Pearce et al., 2020; Petersen et al., 2014). Interestingly, supplementation with the soluble fiber pectin prior to ionizing radiation (IR)-induced intestinal epithelial injury promotes regeneration mediated by injury-mobilized +4 position crypt ISCs, as well as the upregulation of ISC genes such as *Dclk1*, *Lgr5*, *Bmi1*, and *Notch1* following IR (Sureban et al., 2015). Nevertheless, the divergent impacts of SCFAs on ISCs are known to be dependent on intestinal regions, SCFA types, and concentrations (Sakata, 1987; Pearce et al., 2020; Malville-Shipan and Fleming, 1992; Boffa et al., 1992).

At a tissue level, a high-fiber diet is known to strengthen the intestinal mucosal barrier by enhancing the production of mucus and antimicrobial peptides (Paone et al., 2022; Suriano et al., 2022; Silveira et al., 2017), which is important for maintaining optimal barrier function. Mice deprived of fiber for 14 days promote the enrichment of mucus-degrading bacteria, accompanied by the thinning of the mucosal barrier and lethal colitis (Desai et al., 2016). Mechanistically, the protective effects of dietary fiber may be due to its suppression of proinflammatory cytokine production. *In vitro*, butyrate administration to the human HT-29 colonic cell line suppresses TNFα-mediated activation of NF-κB (Inan et al., 2000). Supporting this, UC patients treated with butyrate enemas exhibit reduced macrophage NF-κB activity and disease severity versus placebo, supporting anti-inflammatory roles (Luhrs et al., 2002). As mentioned previously, high-sugar diets limit SCFA production, which in turn increases inflammation and impairs barrier function through suppression of NF-κB (Parada Venegas et al., 2019). Notably, propionate and butyrate also exert tumor-suppressive effects on CRC cell lines through the activation of GPR43, which is significantly downregulated in colorectal tumors (Tang et al., 2011). Butyrate restricts CRC cell proliferation by modulating gene expression of crucial cell cycle regulators (Archer et al., 1998; Hu et al., 2011; Tailor et al., 2014). Importantly, resistant starches reduced tumor burden in an AOM/DSS rat model alongside heightened SCFAs and GPR43 mRNA expression and restricted proliferation and inflammation (Hu et al., 2016). The tumor-suppressive activity of propionate and butyrate on CRC cells *in vitro* has been shown to be mediated by GPR43. Paradoxically, butyrate-induced apoptosis in human CRC cells is correlated with an upregulation of Wnt signaling, which was previously reported as tumor-promoting signaling (Bordonaro et al., 1999; Zeng et al., 2014; Imkeller et al., 2022; Bordonaro et al., 2008), suggesting Wnt-dependent tumor-suppressive actions of SCFA-induced activation of GPR43. Together, these studies suggest the anti-inflammation and anti-cancer effects of a high-fiber diet; however, more mechanistic research is needed on specific mechanisms influencing ISC dynamics in the contexts of inflammation and tumorigenesis.

#### 3.2.2 Vitamin D

Recent research has shown associations between vitamin D and both CRC and IBD. Vitamin D is an important nutrient and hormone for calcium metabolism and mineral homeostasis, but also has roles in inhibiting angiogenesis, activating apoptosis, and displaying immune-modulating effects (Li et al., 2023). Vitamin D is obtained in different forms from dietary sources such as vegetables, dairy, and fish and is also synthesized in the skin from sunlight. Using the UK Biobank Project, Li et al. confirmed an inverse relationship between increased serum 25-hydroxyvitamin D (25(OH)D) and CRC risk (Li et al., 2023). Likewise, for IBD, vitamin D deficiency is highly prevalent among IBD patients, and those with lower levels have worsened disease activity (Vernia et al., 2022). This association is likely due to the immune-regulatory nature of vitamin D as well as through intestinal epithelial integrity, as vitamin D assists in the regulation of tight junctions and the release of antimicrobial peptides such as defensins (Vernia et al., 2022). Vitamin D is a complex hormone, but there is a clear relationship between deficiency and increased risk of IBD and CRC.

Vitamin D signaling profoundly impacts ISC behavior across numerous contexts. Vitamin D derived from sunlight or diet is transformed into metabolically active calcitriol via hydroxylation, which elicits its effects through the vitamin D receptor (VDR). VDRs are expressed in *Lgr5+* stem cells in both normal and tumor human colons, indicating direct regulatory roles (Fernandez-Barral et al., 2020). In homeostasis, calcitriol suppresses human colonic ISC proliferation and assists in maintaining an undifferentiated state by upregulating stemness genes. Calcitriol also reduces normal organoid proliferation without affecting Wnt genes, instead activating LRIG1, a quiescent stem cell marker (Fernandez-Barral et al., 2020). Vitamin D deficiency conversely reduces *Lgr5+* ISC numbers and alters gene expression, increasing absorptive markers and expanding activity of the Wnt signaling pathway in the small intestinal villi and colonic crypts (Peregrina et al., 2015). Vitamin D enhances ISC migratory and proliferative capacity, accelerating differentiation and epithelial repair after experimental colitis (Xu et al., 2021). Future studies should focus on understanding the signaling pathways by which vitamin D exerts its pro-regenerative effects. Vitamin D supplementation has also been shown to maintain barrier integrity by regulating tight junction expression, thereby preventing bacterial translocation (Clark and Mach, 2016). In a murine model of UC using 5% DSS, vitamin D administration attenuates disease severity, as evidenced by enhanced expression of β-catenin and KRT20, indicating accelerated progenitor stem cell activation and mature enterocyte production along the crypt axis (Wibowo et al., 2021). Clinically, vitamin D deficiency is associated with heightened IBD risk and complications, warranting supplementation (Gubatan et al., 2019; Lim et al., 2005; Harries et al., 1985; Guo et al., 2022). In the context of tumorigenesis, VDR has been shown to be overexpressed in early-stage CRC (Matusiak et al., 2005). Instead of its homeostatic function of restricting differentiation, in patient-derived tumor organoids, calcitriol induces differentiation and variably suppresses cancer stem cell proliferation (Fernandez-Barral et al., 2020). Vitamin D supplementation in CRC cells has been shown to block β-catenin transcriptional activity by inducing binding of VDR to β-catenin, thereby preventing β-catenin nuclear translocation (Palmer et al., 2001; Egan et al., 2010). *In vivo*, vitamin D restricts polyp number and tumor load in *Apc* ^min/+^ mice, which has been correlated with reduced expression of β-catenin target genes (Xu et al., 2010). Interestingly, in an AOM/DSS model, vitamin D administration reduced tumor formation yet also inhibited colonic proliferation, corresponding with dampened β-catenin signaling (Xin et al., 2017). These contradictory results may relate to distinctions between inflammatory versus neoplastic contexts.

### 3.3 Nutritional determinant of ISC fate as the common root of diet-related IBD and CRC

In line with the findings mentioned above, western-style diets (WSDs), which are typically characterized by high fat, high sugar and carbohydrate intake, high calories, low fiber, and low vitamin D, appear to be the common root of diet-related IBD and CRC. The pro-tumorigenic effect of WSDs has been well-demonstrated in human CRC and in mice (Arima et al., 2022; Mehta et al., 2017; Newmark et al., 2001; Benninghoff et al., 2020) (Table 2). Specifically, NWD1, a purified rodent WSD characterized by lower levels of vitamin D3, calcium, methyl donors, and fiber, has been shown to disrupt the maturation of ISCs, which reduced the contribution of *Lgr5+* ISCs to homeostasis while reprogramming *Bmi1+* ISCs to acquire stem-like features in the murine intestinal crypts (Li et al., 2019). This reprogramming is characteristic of replicative damage (Schwitalla et al., 2013), which can be partially rescued by additional supplementation of vitamin D and calcium in the diet (Li et al., 2019). Of note, in APC tumor mouse models, NWD1 administration alters cell composition in the crypt-villus axis within the intestinal mucosa even prior to the development of tumors. In the villi, WSD-induced ectopic expression of Paneth cell markers and increased Wnt signaling, which is otherwise restricted to the crypt base (Wang et al., 2011). WSDs are also known to transcriptionally prime the gene expression of colonic mucosa to a higher Wnt or APC-loss tumorigenic state, and supplementation of calcium and vitamin D3 has been shown to prevent these gene expression changes (Yang et al., 2008). In an AOM/DSS mouse model, transitioning to a “healthier” diet consisting of proper micronutrients and fiber has been shown to rectify cell proliferation, increase vitamin D catabolizing enzyme, render *c-myc* and *Axin2* expression under the Wnt pathway, and mitigate the detrimental effects of a WSD in mice (Groschel et al., 2019). In this study, mice were fed either WSD or a normal “healthy” AIN93G diet for 5 weeks before AOM injection and DSS cycles. Mice fed exclusively a WSD exhibited increased inducible nitric oxide synthase, a target of NF-κB that is often highly expressed in the colon mucosa of IBD patients. This multifaceted response postulates the involvement of dietary factors in Wnt and NF-κB signaling in a colitis-associated CRC model (Groschel et al., 2019). Mechanistic studies investigating the regulatory role of WSD on ISC signaling and IBD development are relatively incomplete. Nevertheless, existing mechanistic studies focusing on isolated components of typical WSD support the notion that high calories from fat and sugar intake and low vitamin D and fibers have a profound impact on tissue inflammation, rendering mice more susceptible to DSS-induced colitis (Table 3) (Lee et al., 2017; Burr et al., 2023; Laffin et al., 2019; Shen et al., 2021; Martinez-Medina et al., 2014; Macia et al., 2015; Lagishetty et al., 2010; Zhao et al., 2020). Notably, although the signaling underlying the ISC activities remains unclear, the consumption of red meat, a common component of WSD, has been shown to promote heme-induced epithelial cytotoxicity and hyperproliferation and perturb the mucus barrier function mediated by mucin-degrading bacteria (e.g., *Akkermansia*) (Ijssennagger et al., 2015). High-red meat consumption is also known to alter the gut microbiota to increase colitis and inflammatory cytokine secretion in DSS-treated mice, leading to the reduction of epithelial tight junction proteins and impairment of the colonic barrier (Li D. P. et al., 2021).

**TABLE 2:** Nutritional determinant of intestinal stem cell fate in the diet-related CRC.

Western-style diet				
Dietary model	NWD1			WSD (ssniff EF R/M acc. TD88137 mod.)
Nutritional determinant	High fat, low fiber, low vitamin D			
Disease model	WT	APC-loss	APC-loss	AOM/DSS
ISC consequences	Reduced *Lgr5*+ ISCs, induced *Bmi1*+ ISCs	Dysregulated crypt-villus axis, ectopic expression of Panel cells, increased Wnt activity	Wnt activation, altered metabolic gene expression	Dysregulated cell proliferation, dysregulated Wnt and NF-κB activation
References	Li et al. (2019)	Wang et al. (2011)	Yang et al. (2008)	Groschel et al. (2019)

**TABLE 3:** Nutritional component of Western-style diets and their impact on the intestinal epithelium.

Nutritional component	Impact on intestinal epithelium	References
Fat	Decreases goblet and Paneth cell populations, disrupts gut barrier integrity, increases inflammation, alters intestinal microbiota composition	Lee et al. (2017), Shen et al. (2021)
Sugar	Reduces proliferation of *Lgr5+* ISCs, alters metabolic gene expression, impairs regenerative response to injury, exacerbates DSS-induced inflammation, compromises crypt structure	Burr et al. (2023), Laffin et al. (2019), Martinez-Medina et al. (2014)
Fiber deficiency	Induces dysbiosis, upregulates inflammatory gene expression, increases susceptibility to DSS-induced colitis	Shen et al. (2021), Macia et al. (2015)
Vitamin D deficiency	Increases susceptibility to DSS-induced colitis, reduces antimicrobial protein ANG4 expression, enhances bacterial infiltration	Lagishetty et al. (2010)

Contrary to the widely accepted notion that the WSD worsens IBD, one study demonstrated that a WSD with a high fat content offered protection against DSS-induced colonic inflammation as compared to a low-fat diet (Papoutsis et al., 2022). This unforeseen result has led to a request for additional research into the intricate roles of fat and other nutrients, such as cholesterol, in influencing these outcomes. The authors emphasized the methodological discrepancies in prior studies, specifically pointing out the insufficiency of typical low-fat diets employed as controls due to their high dietary fiber content. In contrast, WSDs typically utilize metabolically inactive cellulose as their source of fiber, resulting in a discrepancy in fiber content. This discrepancy may contribute to the negative consequences associated with WSDs, presumably resulting from diets lacking in dietary fiber, which leads to a less varied bacterial composition and the growth of bacteria that damage the intestinal barrier (Papoutsis et al., 2022).

## 4 Conclusion

**FIGURE 4:**
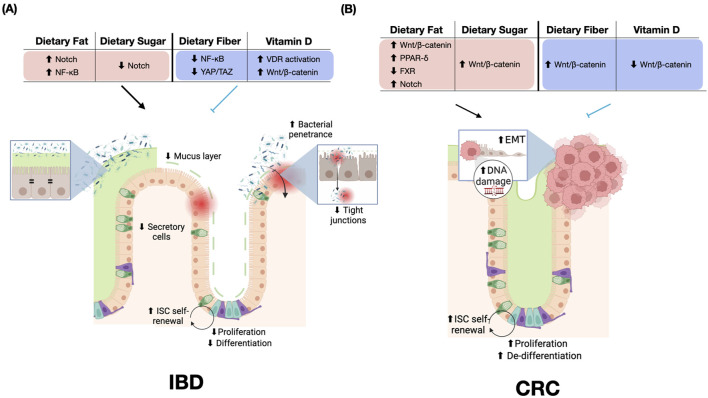
Dietary Effects on Inflammatory Bowel Disease (IBD) and Colorectal Cancer (CRC). **(A)** Impact of dietary components on IBD pathogenesis. Dietary fat increases Notch and NF-κB signaling, while dietary sugar decreases Notch activity. Dietary fiber reduces NF-κB and YAP/TAZ signaling. Vitamin D activates VDR and Wnt/β-catenin pathways. These dietary influences contribute to an impaired mucus layer, reduced secretory cells, increased bacterial penetrance, and compromised tight junctions. ISC self-renewal, proliferation, and differentiation are increased in IBD. **(B)** Effects of dietary components on CRC development. Dietary fat inhibits FXR and activates multiple pathways, including Wnt/β-catenin, PPAR-δ, and Notch. Dietary sugar and fiber both increase Wnt/β-catenin signaling, while vitamin D decreases Wnt/β-catenin signaling. It is important to note that while Wnt/β-catenin signaling is prominently featured, the cellular phenotypes observed in CRC result from the complex interplay of multiple activated pathways, including PPAR-δ and FXR, rather than Wnt/β-catenin signaling alone. These dietary influences in CRC are associated with increased EMT, DNA damage, ISC self-renewal, proliferation, and de-differentiation. The figure highlights the distinct molecular and cellular responses to similar dietary components in IBD versus CRC, emphasizing the context-dependent effects of nutrition on intestinal health and disease progression. Red = disease-promoting, blue = disease-preventing. Abbreviations: ↑, increased; ↓, decreased; EMT, epithelial-mesenchymal transition; ISC, intestinal stem cell; VDR, Vitamin D receptor; NF-κB, Nuclear factor kappa B; PPAR, Peroxisome proliferator-activated receptor; FXR, Farnesoid X receptor; YAP/TAZ, Yes-associated protein/transcriptional co-activator with PDZ-binding motif.

Inflammatory bowel disease (IBD) and colorectal cancer (CRC) are two major gastrointestinal diseases that are not fully explained by genetics and are closely linked to dietary factors. Despite the strong association between IBD and CRC, the role of diet in this relationship has not been thoroughly investigated. Our review integrates the clinical and pre-clinical findings that suggest certain macronutrients, such as dietary fat and sugar, play a significant role in promoting intestinal inflammation and tumorigenesis. Conversely, certain micronutrients, such as fiber and vitamin D, have anti-inflammatory and anti-tumorigenic effects. More importantly, through insights from nutrigenomic studies of ISCs, we have identified key transcription factors responsible for the diet-induced effects on ISC activities, mediating IBD and CRC development. Specifically, we found that dietary fat promotes inflammation and tumorigenesis through the activation of NF-kB and Wnt/β-catenin, respectively. The former can be inhibited by dietary fiber, while the latter is prevented by vitamin D, supporting the disease-prevention potential observed in human studies. Notably, dietary sugar appears to induce inflammation by dampening Notch signaling, a pathway activated by dietary fat and not reversed by either fiber or vitamin D. Together, these findings reveal key dietary factors and the corresponding transcriptional programs that explain ISC-mediated disease phenotypes, highlighting a nutrient-specific, context-dependent gene regulatory network underlying diet-related IBD and CRC (Figure 4).

The complex interactions among these nutrient-sensing transcription factors determine the delicate balance between self-renewal, differentiation, and tumor initiation. Understanding these nutrigenomic pathways offers promising avenues for developing novel diagnostic and therapeutic approaches. Recent technological advances have afforded the ability to perform single-cell RNA sequencing on large-scale levels, as evident by the Gut Cell Atlas, in which hundreds of thousands of cells are mapped and publicly available for analysis, showing transcriptional uniqueness among healthy and diseased gastrointestinal tracts (Elmentaite et al., 2020; Zilbauer et al., 2023). Through the use of single-cell RNA sequencing technology, the development of biomarkers for disease through a nutrigenomics approach is a worthy goal. Profiling stem cell transcriptional landscapes in response to dietary factors may yield new biomarkers for disease risk and progression, while targeting diet-responsive pathways could potentially enhance regeneration, ameliorate inflammation, or suppress tumor formation.

In addition to the dietary factors summarized in the current review, there are dietary patterns, such as the ketogenic diet and low-calorie diets, that show protective benefits through ISC regulation, although complementary human data is currently limited. Ketogenic diets have shown anti-cancer effects in mouse CRC studies, with mixed results regarding inflammation (Zhang et al., 2020; Kong et al., 2021; Li S. et al., 2021). Fasting mimicking diets, a form of low-calorie diet, may partially alleviate IBD symptoms in rodent studies (Rangan et al., 2019), and clinical trials are underway to examine their effects on inflammation in individuals with IBD, addressing the need to comprehend how food intake influences ISCs and contributes to disease. On the contrary, certain dietary practices, such as exclusive enteral nutrition (EEN) for IBD, have shown clinical benefits, particularly in pediatric Crohn’s disease, where randomized clinical trials indicate superior mucosal healing compared to steroid treatment (Borrelli et al., 2006). However, the mechanisms underlying the benefits of EEN remain unclear. Further research into nutrigenomics and transcriptional regulation, as discussed in this review, would be valuable in elucidating these effects.

Future research should focus on elucidating the precise mechanisms by which specific nutrients and metabolites interact with stem cell regulatory networks. Large-scale human studies integrating dietary data, stem cell profiling, and disease outcomes are needed to validate findings from animal models. Additionally, investigating how dietary interventions might be tailored to individual genetic backgrounds could advance personalized prevention and treatment strategies for CRC and IBD. This review highlights the extensive preclinical data that can guide the development of clinical biomarkers and eventual therapeutic interventions. It underscores the significance of diet-dependent predisposition to IBD and CRC development and raises the possibility of targeting ISC nutrigenomics to develop more effective strategies for maintaining gut health and combating the rising global burden of diet-related gastrointestinal diseases.
