# Supplementary material for: Nutrigenomic underpinnings of intestinal stem cells in inflammatory bowel disease and colorectal cancer development
Source: Front Genet. 2024 Aug 30;15:1349717. doi: 10.3389/fgene.2024.1349717 (PMC11393785; doi:10.3389/fgene.2024.1349717)
Supplement: Supplementary file 1 [file Table1.DOCX]

**Box I. Transcription factors that regulate intestinal stem cell function**

**Tissue homeostasis**

In homeostatic conditions, rapidly proliferating *Lgr5+* crypt base columnar cells (CBCs) give rise to bi-lineage potential progenitors, which in turn generate all epithelial lineages to renew and maintain the epithelial barrier [1; 2]. As progenitor cells differentiate from intestinal stem cells (ISCs), they migrate from the crypt base up the intestinal villus or upper crypt of the small intestine or colon, respectively. However, a subset of terminally differentiated secretory cells, namely Paneth cells and deep-crypt secretory cells, migrate downward to reside at the crypt base [3; 4; 5]. *Lyz1*+ Paneth cells function as a key niche cell population for ISC in the small intestine, while *Reg4+* deep crypt secretory cells fulfill this role in the colon [4; 5]. Together with the mesenchymal cell population surrounding the intestinal crypt, the crypt base environment constitutes a permissive niche regulating ISC function in self-renewal and differentiation. The homeostatic tissue-renewing function of ISC is tightly regulated by several core transcriptional pathways: Wnt, Notch, and Hippo (YAP/TAZ).

The canonical Wnt pathway is a key regulator of ISC self-renewal and proliferation. Wnt ligands secreted by crypt base cells bind to Frizzled receptors on ISCs, leading to the stabilization and nuclear translocation of β-catenin, which acts on transcriptional co-factors of Wnt target genes, including LGR5. In the absence of Wnt, β-catenin is phosphorylated and undergoes proteasomal degradation, precluding nuclear entry. Consequently, niche-derived Wnt ligands form a gradient along the crypt-villus axis, with maximal activity at the base, therefore maintaining ISCs in an undifferentiated, proliferative state by activating Wnt target genes. Animal models revealed the essential role of Wnt signaling in maintaining ISC stemness and the undifferentiated state of ISCs; conditional ablation of the β-catenin gene in mice led to the loss of crypts and a decrease in proliferation, with forced differentiation of ISCs into enterocytic lineage [6]. Systemic inhibition of Wnt ligand secretion and therefore the Wnt pathway resulted in reduced proliferative activity in both the small intestine and colonic crypts, in addition to crypt loss combined with the disappearance of stem cell markers [7].

Notch signaling regulates ISC function through its impact on survival, proliferation, and differentiation, particularly absorptive-secretory cell fate decisions [8]. Inhibition of the Notch pathway using dibenzazepine (DBZ) in mice reduced stem cell numbers through reduced proliferative activity and increased apoptosis. This is coupled with the expansion of all secretory cell types [9].

The evolutionarily conserved Hippo cascade has been implicated in maintaining the ISC epithelium. Briefly, activation of the Hippo pathway occurs through a kinase cascade leading to cytoplasmic localization and inactivation of the YAP/TAZ transcriptional coactivator and TEAD, consequently blocking the induction of gene transcriptional programs involved in ISC proliferation and cell survival [10]. In intestinal homeostasis, YAP/TAZ expression is elevated in the crypt compartment [11]. Knockdown of YAP/TAZ resulted in the downregulation of genes comprising ISC signatures and a decrease in goblet cells, possibly through decreased Notch expression, which is rescued by the administration of shRNA-resistant YAP [10]. Deletion of Mst1/Mst2, kinases that inhibit YAP/TAZ nuclear entry, also resulted in the expansion of cells displaying ISC markers accompanied by an increase in *Ki67+* cells and the loss of secretory cells [12].

There is growing evidence that inflammatory signaling can influence ISC regulation. In the intestinal epithelium, the immunomodulatory transcription factor NF-κB cooperates with downstream Wnt and Sox transcription factors to guide Paneth and goblet cell fate decisions [13]. NF-κB signaling can be triggered by several stimuli, such as tumor necrosis factor-alpha (TNFα), interleukin-1 (IL-1), and bacterial lipopolysaccharides [14]. In the small intestine, *Lgr5+* cells constitutively activate NF-κB [15]. Elevated NF-κB activity is also evident in Paneth cells; genetic inhibition of NF-κB in mice resulted in a reduction in Paneth cells and an increase in goblet cells, highlighting the role of the transcription factor in balanced Paneth vs goblet cell differentiation [13]. NF-κB inhibitor IκBα deficiency led to aberrant NF-κB activity and subsequently influenced cell differentiation within the intestinal epithelium, evidenced by the absence of *Lyz1+* Paneth cells and a decrease in alcian blue-positive goblet cells, as well as an ISC phenotype reminiscent of fetal stages [16]. Furthermore, NF-κB works cooperatively with Wnt signaling to affect the expansion of crypt stem cells through the dedifferentiation of non-stem cells [17].

**Post-injury regeneration**

The intestinal epithelial barrier is susceptible to injury from insults such as infection, irradiation, and ischemia. In the process of post-injury regeneration, CBCs can become sensitive and depleted, leading to the activation of reserve stem cell populations. Traditionally, reserve stem cells have been primarily attributed to +4 position cells located just above the CBCs, characterized by their quiescent nature and expression of specific markers such as *Bmi1*, *Hopx*, *Tert*, *Krt19*, *Clu*, *Mex3a*, and *Lrig1*. Studies have highlighted their crucial role during epithelial injury and regeneration, as they display self-renewal potential and heightened activity in response to tissue damage [18]. Yet recent lineage-tracing experiments have illuminated the plasticity and dedifferentiation between ISCs and progenitor populations during periods of inflammation and regeneration [19; 20; 21; 22]. Within this dynamic environment, even short-lived progenitors and a subset of mature cells can dedifferentiate, reverting to a stem cell state and assuming the role of an alternative source of ISC.

Wnt, Notch, and Hippo become activated upon injury. These transcription factors oversee the balance between ISC differentiation versus dedifferentiation, as well as promote the proliferation needed for tissue regeneration. Specifically, the Wnt pathway elicits cellular plasticity, enabling intestinal epithelial regeneration. Organoid models have revealed that Wnt proteins sustain ISC multipotency while also directing the dedifferentiation of secretory progenitors back into stem-like states [23]. In murine models, the administration of R-spondin, which potentiates Wnt signaling, mitigated architectural damage induced by cytotoxic insults from dextran sulfate sodium (DSS), through an increase in crypt proliferation and goblet cell number [24; 25]. Activation of Wnt ligands and target genes was associated with Paneth cell dedifferentiation in response to DSS treatment [26]. Further, genetic ablation of the ISC-restricted transcription factor and Wnt target gene, *Ascl2,* has been suggested to be critical for ISC restoration after irradiation or ablation [27]. YAP/YAZ are engaged in the complex process of epithelial tissue renewal, serving as targets of Wnt signaling critical for regeneration and regulating the transcription of genes associated with proliferation, cell survival, cell fate, and aiding *Lgr5+* ISC pool recovery and crypt regeneration [28; 29; 30; 31]. Increased YAP activity was evident after DSS-induced damage in colonic crypts, and its deletion impaired regeneration [29]. In a DSS-induced colonic regeneration mouse model, YAP/TAZ was attributed to the reprogramming of the intestinal epithelium to a fetal-like state, with the emergence of fetal markers and suppression of adult stem and differentiated cell markers [32]. YAP/TAZ also regulates Wnt [33]; increased YAP levels after injury dampen Wnt signals, curbing Paneth cell formation and ISC expansion. YAP also modulates Wnt signaling by preventing aberrant hyperactivation [28]. Additionally, the upregulation of Notch signaling maintains ISC proliferative capacity after insult while enabling secretory progenitor dedifferentiation [34; 35; 36]. Moreover, Notch controls the interconversion and asymmetric division of *Lgr5*+ and *Bmi1+* ISCs vital for post-injury regeneration [37]. DSS treatment increased the asymmetric division frequency and Notch signaling, while administration of Notch inhibitor DAPT reversed these effects. Furthermore, suppression of Notch reduced the survival of *Dclk1+* reserve stem cells following irradiation [38].

The NF-κB pathway responds to microenvironment perturbations to regulate barrier immunity [39]. Proper functioning of the intestinal barrier, orchestrated by NF-κB, is not only important for ISC homeostasis, but heightened intestinal permeability can disrupt tissue balance. Upon detecting the detachment of intestinal epithelial cells, this signaling pathway is activated, diminishing apoptosis to uphold homeostasis. In addition, it instigates heightened cytokine production, thereby expediting the regenerative process in cases of intestinal injuries [40].

**References**

[1] N. Barker, J.H. van Es, J. Kuipers, P. Kujala, M. van den Born, M. Cozijnsen, A. Haegebarth, J. Korving, H. Begthel, P.J. Peters, and H. Clevers, Identification of stem cells in small intestine and colon by marker gene Lgr5. Nature 449 (2007) 1003-7.

[2] A.J.M. Santos, Y.H. Lo, A.T. Mah, and C.J. Kuo, The Intestinal Stem Cell Niche: Homeostasis and Adaptations. Trends Cell Biol 28 (2018) 1062-1078.

[3] S.R. Lueschow, and S.J. McElroy, The Paneth Cell: The Curator and Defender of the Immature Small Intestine. Front Immunol 11 (2020) 587.

[4] C. Wallaeys, N. Garcia-Gonzalez, and C. Libert, Paneth cells as the cornerstones of intestinal and organismal health: a primer. EMBO Mol Med 15 (2023) e16427.

[5] H. McCarthy, and S. Keely, Re-evaluating the Role of Deep Crypt Secretory Cells in Intestinal Homeostasis. Cell Mol Gastroenterol Hepatol 15 (2023) 1020-1021.

[6] T. Fevr, S. Robine, D. Louvard, and J. Huelsken, Wnt/beta-catenin is essential for intestinal homeostasis and maintenance of intestinal stem cells. Mol Cell Biol 27 (2007) 7551-9.

[7] T. Valenta, B. Degirmenci, A.E. Moor, P. Herr, D. Zimmerli, M.B. Moor, G. Hausmann, C. Cantu, M. Aguet, and K. Basler, Wnt Ligands Secreted by Subepithelial Mesenchymal Cells Are Essential for the Survival of Intestinal Stem Cells and Gut Homeostasis. Cell Rep 15 (2016) 911-918.

[8] B. Zhou, W. Lin, Y. Long, Y. Yang, H. Zhang, K. Wu, and Q. Chu, Notch signaling pathway: architecture, disease, and therapeutics. Signal Transduct Target Ther 7 (2022) 95.

[9] K.L. VanDussen, A.J. Carulli, T.M. Keeley, S.R. Patel, B.J. Puthoff, S.T. Magness, I.T. Tran, I. Maillard, C. Siebel, A. Kolterud, A.S. Grosse, D.L. Gumucio, S.A. Ernst, Y.H. Tsai, P.J. Dempsey, and L.C. Samuelson, Notch signaling modulates proliferation and differentiation of intestinal crypt base columnar stem cells. Development 139 (2012) 488-97.

[10] M. Imajo, M. Ebisuya, and E. Nishida, Dual role of YAP and TAZ in renewal of the intestinal epithelium. Nat Cell Biol 17 (2015) 7-19.

[11] F.D. Camargo, S. Gokhale, J.B. Johnnidis, D. Fu, G.W. Bell, R. Jaenisch, and T.R. Brummelkamp, YAP1 increases organ size and expands undifferentiated progenitor cells. Curr Biol 17 (2007) 2054-60.

[12] D. Zhou, Y. Zhang, H. Wu, E. Barry, Y. Yin, E. Lawrence, D. Dawson, J.E. Willis, S.D. Markowitz, F.D. Camargo, and J. Avruch, Mst1 and Mst2 protein kinases restrain intestinal stem cell proliferation and colonic tumorigenesis by inhibition of Yes-associated protein (Yap) overabundance. Proc Natl Acad Sci U S A 108 (2011) E1312-20.

[13] C. Brischetto, K. Krieger, C. Klotz, I. Krahn, S. Kunz, M. Kolesnichenko, P. Mucka, J. Heuberger, C. Scheidereit, and R. Schmidt-Ullrich, NF-kappaB determines Paneth versus goblet cell fate decision in the small intestine. Development 148 (2021).

[14] E.D. Strauch, B.L. Bass, J.N. Rao, J.A. Vann, and J.Y. Wang, NF-kappaB regulates intestinal epithelial cell and bile salt-induced migration after injury. Ann Surg 237 (2003) 494-501.

[15] S. Lai, R. Cheng, D. Gao, Y.G. Chen, and C. Deng, LGR5 constitutively activates NF-kappaB signaling to regulate the growth of intestinal crypts. FASEB J 34 (2020) 15605-15620.

[16] L. Marruecos, J. Bertran, Y. Guillen, J. Gonzalez, R. Batlle, E. Lopez-Arribillaga, M. Garrido, C. Ruiz-Herguido, D. Lisiero, M. Gonzalez-Farre, S. Arce-Gallego, M. Iglesias, A.R. Nebreda, S. Miyamoto, A. Bigas, and L. Espinosa, IkappaBalpha deficiency imposes a fetal phenotype to intestinal stem cells. EMBO Rep 21 (2020) e49708.

[17] S. Schwitalla, A.A. Fingerle, P. Cammareri, T. Nebelsiek, S.I. Goktuna, P.K. Ziegler, O. Canli, J. Heijmans, D.J. Huels, G. Moreaux, R.A. Rupec, M. Gerhard, R. Schmid, N. Barker, H. Clevers, R. Lang, J. Neumann, T. Kirchner, M.M. Taketo, G.R. van den Brink, O.J. Sansom, M.C. Arkan, and F.R. Greten, Intestinal tumorigenesis initiated by dedifferentiation and acquisition of stem-cell-like properties. Cell 152 (2013) 25-38.

[18] K. Kurokawa, Y. Hayakawa, and K. Koike, Plasticity of Intestinal Epithelium: Stem Cell Niches and Regulatory Signals. Int J Mol Sci 22 (2020).

[19] K.S. Yan, L.A. Chia, X. Li, A. Ootani, J. Su, J.Y. Lee, N. Su, Y. Luo, S.C. Heilshorn, M.R. Amieva, E. Sangiorgi, M.R. Capecchi, and C.J. Kuo, The intestinal stem cell markers Bmi1 and Lgr5 identify two functionally distinct populations. Proc Natl Acad Sci U S A 109 (2012) 466-71.

[20] E.M.F. de Sousa, and F.J. de Sauvage, Cellular Plasticity in Intestinal Homeostasis and Disease. Cell Stem Cell 24 (2019) 54-64.

[21] A. Baulies, N. Angelis, and V.S.W. Li, Hallmarks of intestinal stem cells. Development 147 (2020).

[22] T. Hagemann, F. Balkwill, and T. Lawrence, Inflammation and cancer: a double-edged sword. Cancer Cell 12 (2007) 300-1.

[23] J.H. van Es, T. Sato, M. van de Wetering, A. Lyubimova, A.N. Yee Nee, A. Gregorieff, N. Sasaki, L. Zeinstra, M. van den Born, J. Korving, A.C.M. Martens, N. Barker, A. van Oudenaarden, and H. Clevers, Dll1+ secretory progenitor cells revert to stem cells upon crypt damage. Nat Cell Biol 14 (2012) 1099-1104.

[24] J. Zhao, J. de Vera, S. Narushima, E.X. Beck, S. Palencia, P. Shinkawa, K.A. Kim, Y. Liu, M.D. Levy, D.J. Berg, A. Abo, and W.D. Funk, R-spondin1, a novel intestinotrophic mitogen, ameliorates experimental colitis in mice. Gastroenterology 132 (2007) 1331-43.

[25] C. Harnack, H. Berger, A. Antanaviciute, R. Vidal, S. Sauer, A. Simmons, T.F. Meyer, and M. Sigal, R-spondin 3 promotes stem cell recovery and epithelial regeneration in the colon. Nat Commun 10 (2019) 4368.

[26] M. Schmitt, M. Schewe, A. Sacchetti, D. Feijtel, W.S. van de Geer, M. Teeuwssen, H.F. Sleddens, R. Joosten, M.E. van Royen, H.J.G. van de Werken, J. van Es, H. Clevers, and R. Fodde, Paneth Cells Respond to Inflammation and Contribute to Tissue Regeneration by Acquiring Stem-like Features through SCF/c-Kit Signaling. Cell Rep 24 (2018) 2312-2328 e7.

[27] K. Murata, U. Jadhav, S. Madha, J. van Es, J. Dean, A. Cavazza, K. Wucherpfennig, F. Michor, H. Clevers, and R.A. Shivdasani, Ascl2-Dependent Cell Dedifferentiation Drives Regeneration of Ablated Intestinal Stem Cells. Cell Stem Cell 26 (2020) 377-390 e6.

[28] A. Gregorieff, Y. Liu, M.R. Inanlou, Y. Khomchuk, and J.L. Wrana, Yap-dependent reprogramming of Lgr5(+) stem cells drives intestinal regeneration and cancer. Nature 526 (2015) 715-8.

[29] J. Cai, N. Zhang, Y. Zheng, R.F. de Wilde, A. Maitra, and D. Pan, The Hippo signaling pathway restricts the oncogenic potential of an intestinal regeneration program. Genes Dev 24 (2010) 2383-8.

[30] P. Karpowicz, J. Perez, and N. Perrimon, The Hippo tumor suppressor pathway regulates intestinal stem cell regeneration. Development 137 (2010) 4135-45.

[31] E.R. Barry, T. Morikawa, B.L. Butler, K. Shrestha, R. de la Rosa, K.S. Yan, C.S. Fuchs, S.T. Magness, R. Smits, S. Ogino, C.J. Kuo, and F.D. Camargo, Restriction of intestinal stem cell expansion and the regenerative response by YAP. Nature 493 (2013) 106-10.

[32] S. Yui, L. Azzolin, M. Maimets, M.T. Pedersen, R.P. Fordham, S.L. Hansen, H.L. Larsen, J. Guiu, M.R.P. Alves, C.F. Rundsten, J.V. Johansen, Y. Li, C.D. Madsen, T. Nakamura, M. Watanabe, O.H. Nielsen, P.J. Schweiger, S. Piccolo, and K.B. Jensen, YAP/TAZ-Dependent Reprogramming of Colonic Epithelium Links ECM Remodeling to Tissue Regeneration. Cell Stem Cell 22 (2018) 35-49 e7.

[33] L. Azzolin, T. Panciera, S. Soligo, E. Enzo, S. Bicciato, S. Dupont, S. Bresolin, C. Frasson, G. Basso, V. Guzzardo, A. Fassina, M. Cordenonsi, and S. Piccolo, YAP/TAZ incorporation in the beta-catenin destruction complex orchestrates the Wnt response. Cell 158 (2014) 157-70.

[34] A.J. Carulli, T.M. Keeley, E.S. Demitrack, J. Chung, I. Maillard, and L.C. Samuelson, Notch receptor regulation of intestinal stem cell homeostasis and crypt regeneration. Dev Biol 402 (2015) 98-108.

[35] R. Okamoto, K. Tsuchiya, Y. Nemoto, J. Akiyama, T. Nakamura, T. Kanai, and M. Watanabe, Requirement of Notch activation during regeneration of the intestinal epithelia. Am J Physiol Gastrointest Liver Physiol 296 (2009) G23-35.

[36] S. Yu, K. Tong, Y. Zhao, I. Balasubramanian, G.S. Yap, R.P. Ferraris, E.M. Bonder, M.P. Verzi, and N. Gao, Paneth Cell Multipotency Induced by Notch Activation following Injury. Cell Stem Cell 23 (2018) 46-59 e5.

[37] T. Srinivasan, E.B. Than, P. Bu, K.L. Tung, K.Y. Chen, L. Augenlicht, S.M. Lipkin, and X. Shen, Notch signalling regulates asymmetric division and inter-conversion between lgr5 and bmi1 expressing intestinal stem cells. Sci Rep 6 (2016) 26069.

[38] D. Qu, R. May, S.M. Sureban, N. Weygant, P. Chandrakesan, N. Ali, L. Li, T. Barrett, and C.W. Houchen, Inhibition of Notch signaling reduces the number of surviving Dclk1+ reserve crypt epithelial stem cells following radiation injury. Am J Physiol Gastrointest Liver Physiol 306 (2014) G404-11.

[39] S.C. Sun, The noncanonical NF-kappaB pathway. Immunol Rev 246 (2012) 125-40.

[40] M. Roulis, C. Nikolaou, E. Kotsaki, E. Kaffe, N. Karagianni, V. Koliaraki, K. Salpea, J. Ragoussis, V. Aidinis, E. Martini, C. Becker, H.R. Herschman, S. Vetrano, S. Danese, and G. Kollias, Intestinal myofibroblast-specific Tpl2-Cox-2-PGE2 pathway links innate sensing to epithelial homeostasis. Proc Natl Acad Sci U S A 111 (2014) E4658-67.
